# Supplementary material for: Coral reef degradation at an atoll of the Western Colombian Caribbean
Source: PeerJ. 2023 Apr 12;11:e15057. doi: 10.7717/peerj.15057 (PMC10105559; doi:10.7717/peerj.15057)
Supplement: Supplemental Information 1 — Average abundance (% cover transformed to root arcosine) of typifying benthic organisms in each group formed by the CLUSTER analysis over the cover of the benthic community considered, their contribution (%) to the within-group similarity, and the cumulative total (%) of contributions (90% cut-off). Hard corals (C; Scleractinia and Milleporidae), fleshy macroalgae (MALG), calcareous macroalgae (CALG), encrusting algae (EALG), octocorals (OCTO) and sponges (SPG). [file peerj-11-15057-s001.pdf]

Table S1. Summary of SIMPER for Quitasueño benthic community. Average abundance (% cover transformed to root arcosine) of typifying benthic organisms in each group formed by the CLUSTER analysis over the cover of the benthic community considered, their contribution (%) to the within-group similarity, and the cumulative total (%) of contributions (90% cut-off). Hard corals (C; Scleractinia and Milleporidae), fleshy macroalgae (MALG), calcareous macroalgae (CALG), encrusting algae (EALG), octocorals (OCTO) and sponges (SPG).

**GROUP A**					
Average similarity: 87,52					
	Average abundance	Av.Diss	Diss/SD	Contrib%	Cum.%
MALG	1,57	49,78	14,65	56,88	56,88
C	0,8	21,07	3,18	24,08	80,96
ESP	0,29	7,57	4,42	8,65	89,61
OCTO	0,34	7,36	1,75	8,41	98,02
**GROUP B**					
Average similarity: 87,66					
MALG	1,57	68,97	9,06	78,68	78,68
C	0,39	12,64	2,05	14,42	93,1
**GROUP C**					
Average similarity: 86,33					
MALG	1,57	48,2	-	55,83	55,83
ESP	0,71	19,42	-	22,5	78,33
C	0,51	13,84	-	16,03	94,36
**GROUP D**					
Average similarity: 84,96					
MALG	1,57	47,02	9,13	55,34	55,34
EALG	0,66	16,81	4,73	19,78	75,12
C	0,68	16,37	4,94	19,26	94,39
**GROUP E**					
Average similarity: 82,67					
MALG	1,57	46,86	23,87	56,68	56,68
C	1,22	33,08	7,31	40,02	96,7
**GROUP F**					
Average similarity: 75,94					
MALG	1,57	40,14	-	52,86	52,86
CALG	0,88	20,07	-	26,43	79,29
C	0,47	5,91	-	7,78	87,07
ESP	0,56	5,76	-	7,59	94,66
**GROUP G**					
Average similarity: 74,51					
C	1,47	31,41	5,03	42,16	42,16

MALG	1,22	23,76	5,07	31,88	74,04
OCTO	0,57	8,76	2,94	11,76	85,8
ESP	0,44	4,74	0,83	6,36	92,16
**GROUP J**					
Average similarity: 73,24					
C	1,57	61,8	5,28	84,38	84,38
MALG	0,31	4,38	0,77	5,98	90,36
**GROUP K**					
Average similarity: 78,73					
EALG	1,57	39,03	10,57	49,57	49,57
MALG	0,91	20,61	12,1	26,17	75,75
C	0,68	14,39	4,83	18,27	94,02
