# Supplementary figures and images for: Coral reef degradation at an atoll of the Western Colombian Caribbean
Source: PeerJ. 2023 Apr 12;11:e15057. doi: 10.7717/peerj.15057 (PMC10105559; doi:10.7717/peerj.15057)

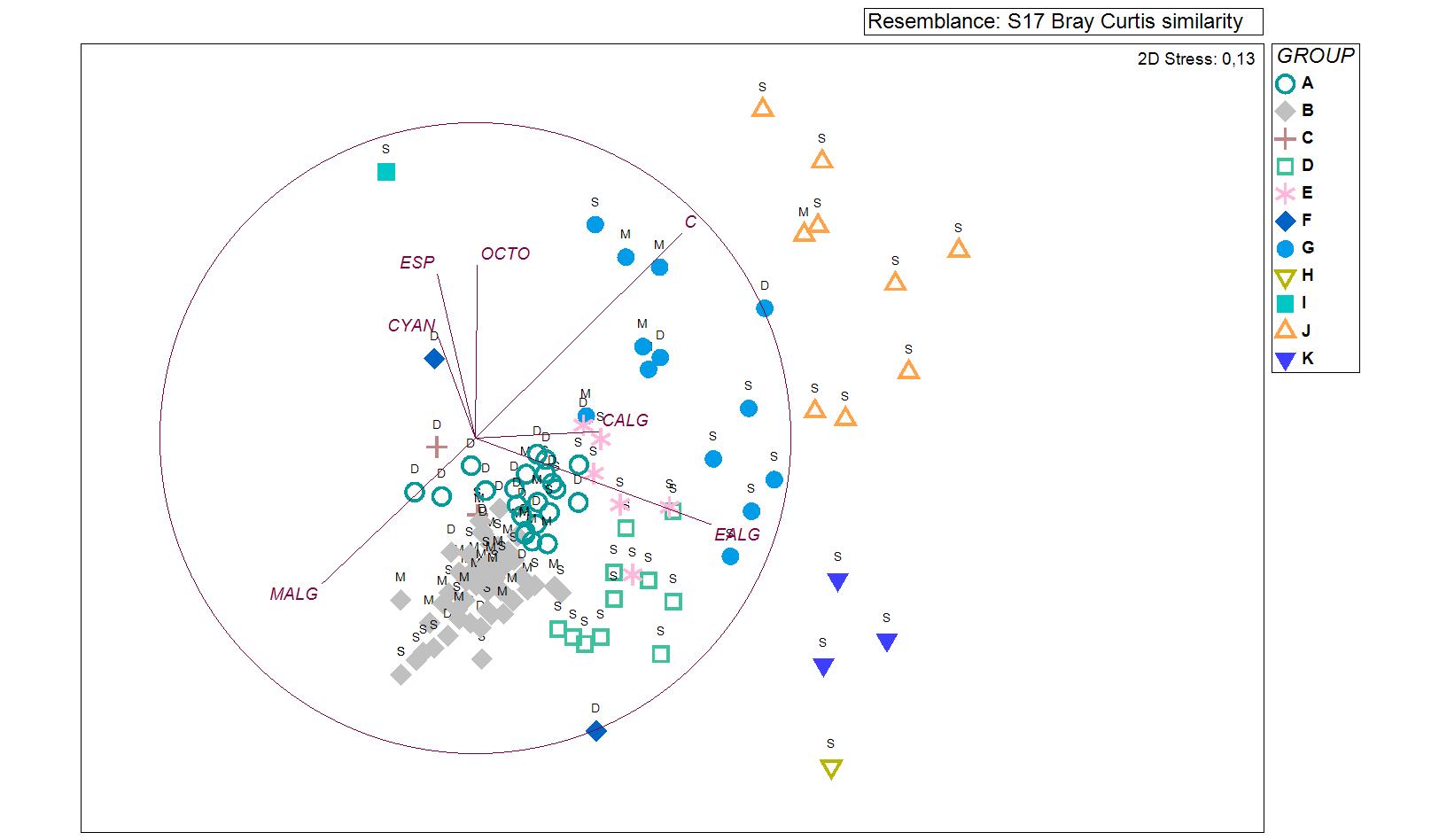

Supplement: Supplemental Information 2 — The vectors show the benthic groups evaluated and the depth range is detailed for each station (S: Shallow [<8 m]; M: Medium [8–12 m] and D: Deep [>12 m]). [file peerj-11-15057-s002.jpg]
